# Observing quantum coherence from photons scattered in free-space

**DOI:** 10.1038/s41377-021-00565-y

**Published:** 2021-06-07

**Authors:** Shihan Sajeed, Thomas Jennewein

**Affiliations:** 1 Institute for Quantum Computing, University of Waterloo, Waterloo, ON N2L 3G1 Canada; 2 Department of Physics and Astronomy, University of Waterloo, Waterloo, ON N2L 3G1 Canada

**Keywords:** Single photons and quantum effects, Quantum optics, Imaging and sensing, Fibre optics and optical communications, Quantum optics

## Abstract

Quantum channels in free-space, an essential prerequisite for fundamental tests of quantum mechanics and quantum technologies in open space, have so far been based on direct line-of-sight because the predominant approaches for photon-encoding, including polarization and spatial modes, are not compatible with randomly scattered photons. Here we demonstrate a novel approach to transfer and recover quantum coherence from scattered, non-line-of-sight photons analyzed in a multimode and imaging interferometer for time-bins, combined with photon detection based on a 8 × 8 single-photon-detector-array. The observed time-bin visibility for scattered photons remained at a high 95% over a wide scattering angle range of −45^0^ to +45^0^, while the individual pixels in the detector array resolve or track an image in its field of view of ca. 0.5°. Using our method, we demonstrate the viability of two novel applications. Firstly, using scattered photons as an indirect channel for quantum communication thereby enabling non-line-of-sight quantum communication with background suppression, and secondly, using the combined arrival time and quantum coherence to enhance the contrast of low-light imaging and laser ranging under high background light. We believe our method will instigate new lines for research and development on applying photon coherence from scattered signals to quantum sensing, imaging, and communication in free-space environments.

## Introduction

Quantum coherence is a key ingredient in many fundamental tests and applications of quantum mechanics including quantum communication^1^, characterization of single-photon sources^2^, generation of non-classical states^3^, quantum metrology^4^, quantum teleportation^5^, quantum fingerprinting^6^, quantum cloning^7^, demonstrating quantum optical phenomena^8^, and quantum computing^9^ etc. The ability to transfer quantum coherence via scattering surfaces and its successful recovery from scattered photons enhances several applications of quantum technologies. For instance, quantum communication capable of operating over a scattering channel could accommodate free space communication with non-line-of-sight between multiple users such as indoors around corners, or with short range links with moving systems. Furthermore, the photon coherence recovered from scattered light could be utilized to improve noise performance in low-light and 3D imaging, non-line-of-sight imaging^10,11^, velocity measurement^12^, light detection and ranging (LIDAR), surface characterization, or biomedical sample identification.

Currently, the predominant photon encoding used on free-space quantum channels is polarization, because it is not impacted by turbulent atmosphere for clear line-of-sight transmission^13^. When photons are scattered, however, their polarization states are inherently disturbed, and the quantum encoding is degraded. A previous study^14^ showed that the observed polarization visibility depends on the scattering surface material, and even the best material (cinematic silver screen) showed a strong dependence on the photon scattering angles with only a total angle of <45° was suitable for quantum communications. Another approach to encode free-space channels is the use of higher-order spatial modes, recently utilized for intracity quantum key distribution^15^, yet these photon states are directly impacted by wavefront distortion and are expected to completely vanish upon random scattering from a surface^16,17^.

Interestingly, time-bin encoding^1,18,19^, – although widely used for single-mode optical fibers^19–23^ – has only been recently demonstrated for free-space channels^24–30^ due to the problem of atmospheric turbulence and scattering. In particular, refs. ^28–30^ solved the problem of atmospheric mode distortion by converting the distorted beam back into a single-mode at the receiver at the expense of additional loss and complexity. Utilization of adaptive optics to correct wavefront could be another solution but it is expensive and technically challenging^31,32^. Refs. ^24–26^ solved the problem by making use of field-widened interferometers at the analyzer setup. However, all these experiments used direct line-of-sight free-space quantum channels and to the best of our knowledge, no experiment has been reported yet that demonstrates reliable transfer and recovery of time-bin states over non-line-of-sight (nLOS) free-space quantum channels.

Here we utilize quantum coherence encoded in time-bins which is robust upon scattering and suitable for our purpose. The multimode states of light have been utilized using field-widened interferometers, or imaging interferometers, thereby solving the wavefront distortions caused by turbulent media. Such interferometers have the additional benefit of preserving an image. We thus implemented an imaging time-bin interferometer equipped with a single-photon-detector-array (SPDA) sensor, with 8 × 8 pixels covering a field of ~0.5°. The photon detector achieves high temporal precision of ≈120 ps, combined with the ability to spatially resolve the field-of-view with excellent time-bin visibility across the whole sensor area. We demonstrate that our system allows imaging a target that was illuminated with photons prepared with specific phase-signatures, that are recovered from the scattered photons with excellent phase coherence by the illuminated pixels. We discuss the viability of our method in the context of two relevant applications.

## Result

### Experiment

**Fig. 1:**
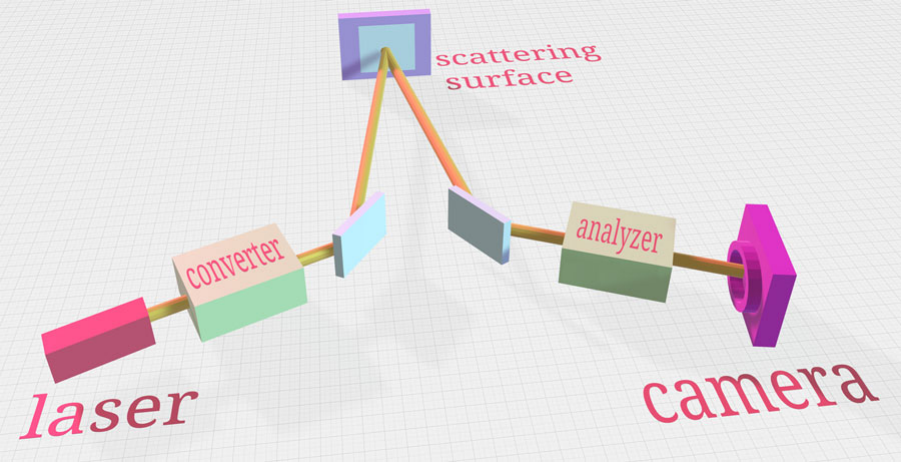
The experimental setup. Optical pulses from a laser are sent through a phase Converter, which creates the initial time-bin states, while the multimode Analyzer measures the signals scattered off the target (regular white paper). A single-photon-detector-array is used as the detection device, with 8 × 8 individual pixels which are time-tagged separately. During the initial alignment, the incident angle = reflected angle = 25°

**Fig. 2:**
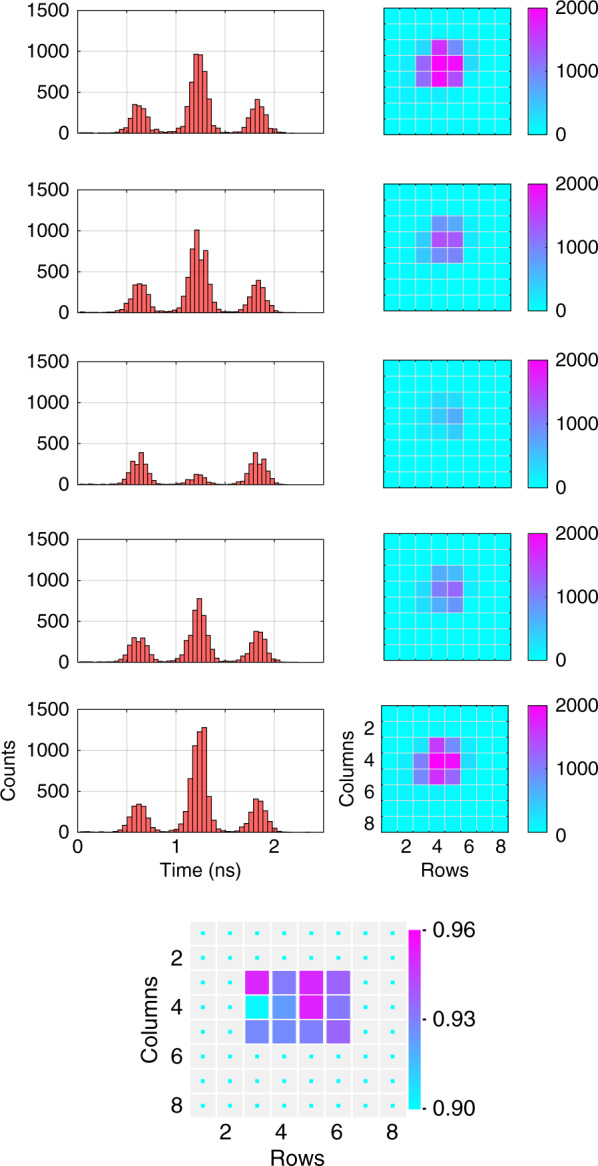
Observed interference. Top left: histograms of the arrival times of photons at pixel number (4,4) (row 4, column 4). The three separate peaks correspond to photons coming via SS (right), SL or LS (middle), and LL (left) paths (see text for more detail). Five different phase-instances are shown. The visibility at this pixel – calculated by curve-fitting – was *V*_(4,4)_ ≈ 0.95. Top right: middle-peak intensity at the illuminated pixels at the corresponding phase-instances. Bottom: visibilities of the illuminated pixels calculated after fitting the curves. The visibilities range from 0.9 to 0.95. Both the color and the size of the *square* marker in each pixel area are indicators of the visibility. Pixel number (1,1) was used as a trigger. The temporal precision was ≈120 ps

The experimental setup is shown in Fig. 1 (see Methods section for additional details). Each pulse from the laser passes through the Converter – an unbalanced Michelson interferometer (UMI) – that converts it into two coherent pulses separated by a time delay according to the path difference ∆_*C*_. These signals are sent towards the target sample covered with a diffusive material (regular white paper) acting as the scattering surface, which can be rotated to vary the angle of incidence. Some of the photons scattered from the surface are captured and guided through the Analyzer, a second UMI with path difference ∆_*A*_. Finally, the photons emerging from the Analyzer are focused into a single-photon-detector-array (SPDA) containing 64 single-photon avalanche photodiodes – hereafter referred to as pixels – arranged in a 8 × 8 row-column configuration, which are free-running and individually time-tagged. Each detected photon could have traversed one of the four possible paths: short-short (SS), short-long (SL), long-short (LS), and long-long (LL) with first (second) letter denoting the path taken inside Converter (Analyzer). The path-differences of the two UMI are tightly matched, i.e., ∆_*C*_ ≈ ∆_*A*_, and interference can be observed, as shown in Fig. 2. Piezoelectric actuators are placed at the short arm of each interferometer to vary their respective phase. To compensate for variable angle of incidence and mode-distortion, a 118-mm-long glass cube with refractive index 1.4525 is placed in the long arm of both interferometers.

A sinusoidal phase difference was introduced between the two interfering (SL and LS) pulses by applying a 0.1-Hz 10 *V* peak-to-peak ramp voltage at the Analyzer piezoelectric actuator (i.e., the length of the LS path was varied with respect to SL). The resultant outcomes are shown in Fig. 2. The top left columns show histograms of detection times – with respect to trigger signal – at one of the central pixels (4,4) (row 4, column 4) for five different phase-instances. The three peaks correspond to photons coming via SS (right), SL or LS (middle), and the LL (left) paths. The temporal precision was ≈120 ps. The measured visibility – calculated by fitting the curve – was *V*_(4,4)_ ≈ 0.95. The right column shows the corresponding middle-peak intensity for other illuminated pixels. In this case, only the events detected during the 0.6-ns window – centered around the middle-peak – were post-selected. The data collection time was 1 s for the left and 0.1 s for the right columns. The visibilities of the illuminated pixels – calculated after fitting the curves – are shown at the bottom which range from 0.9 to 0.95.

We note that while the interference visibility *V* has traditionally been considered as a signature of coherence, there have been cases where – under certain conditions – an enhancement of *V* was observed with increasing decoherence^33,34^, thus creating doubt on its efficacy as a true signature. Furthermore, newer measures of coherence, for example, based on the *l*_1_-norm of the off-diagonal elements of the density matrix of the quanton, has been developed^35–37^ which replaced *V* in the generalized duality relations to capture the correct variation of coherence with decoherence^38^. However, scattering from a ‘smooth’ regular surface ensures that the number of possible interference paths is restricted to two for which the *l*_1_-norm based coherence is identical to the interference visibility *V*^38,39^. Hence, for the rest of this paper, we use the *V* as a measure of coherence. In free-space channels, decoherence mechanism such as dispersion and atmospheric turbulence could lead to a degradation of *V*. However, in our case, turbulence has been taken care of by the imaging (field-widened) interferometers, while for our laser pulse-width of 300 ps, dispersion is negligible^40,41^.

### Robust and stable phase coherence under scattering angle variations

**Fig. 3:**
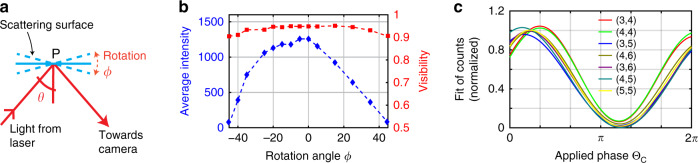
Visibility with scattered light and coherence among the pixels. **a** The incident angle *θ* was varied by rotating the scattering surface about point *P* by an angle *φ* from –45° to +45° while keeping the position of laser and camera constant. **b** Variation of visibility and intensity with rotation angle. Here, rotation angle *φ* = 0° corresponds to incidence angle of *θ* = 25° as shown in Fig. 1. For rotation angles higher than ±45^0^, sufficient amount of photon could not be collected by the camera. The average intensity for each angle is shown on the right axis. The collection time for each data point is 20 s. **c** Variation of counts versus phases applied at the Converter for the illuminated pixels. Pixel number (1,1) was used as a trigger

During the initial alignment, specular reflection was used by setting the incident angle = reflected angle = 25°. We refer to the incident angle as *θ*. Then, using a rotational mount, the scattering surface was rotated about *P* to vary *θ* while keeping the position of the camera fixed (see Fig. 3a). For different rotation angle *φ*, the corresponding average intensity and visibilities are shown in Fig. 3b for pixel number (4,4). For = 25° (*φ* = 0°), the majority of photons collected by the camera was due to specular reflection. As *θ* (*φ*) was varied, the intensity followed the typical scattering pattern consisting of specular and diffusive reflection^42^. At larger rotation angles *φ*, the collected photons were mainly due to scattering from the diffused surface. For *φ* > |45^0^|, the amount of photons collected were too low. However, within the range *φ*∈{–45^0^,+45^0^}, although the number of detected photons varied, the visibility remained fairly constant at ~95% with <10% variation. The same behavior was observed for other illuminated pixels.

### Recovery of coherence while imaging

As we will demonstrate, this approach offers the unique ability for individual pixels in this imaging analyzer to fully detect the coherence, while at the same time resolving spatial modes of the scattered photons. To ensure that the camera received mostly scattered photons, the scattering surface was rotated to set *φ* = 20^0^ (see Fig. 3a) at which the value of visibility was still close to 0.95. Similar to Fig. 2, only the pixels in the center of the sensor were illuminated. By applying a periodic voltage to the piezoelectric actuator in the Converter interferometer, a sinusoidal phase variation was induced among the outgoing time-bin photons. For each phase *θ*_*c*_, the corresponding counts at the illuminated pixels were recorded. No voltage was applied at the Analyzer piezoelectric actuator. The fitted count-versus-phase curves for the illuminated pixels are shown in Fig. 3c. The result verified that although each pixel saw a different set of spatial modes of the incoming light, the phase *θ*_*c*_ was preserved by the modes even after going through diffused scattering and was successfully recovered from the scattered photons by each individual pixel independently. The test was repeated by illuminating all pixels of the SPDA for different scattering angles *φ*, yielding similar results.

### Quantum communication with scattered light

In this section, we demonstrate the viability of free-space non-line-of-sight (NLOS) quantum communication experiments with ‘scattered photons’. Here, nLOS refers to the fact that no direct path exists between sender and receiver; neither do they have access to any specular reflector, i.e., mirror surface, at any intermediate point. In particular, we show that a quantum state of the form $\left. {\left| {\psi _A} \right.} \right\rangle = \left. {\left| e \right.} \right\rangle + e^{i\theta _A}\left. {\left| l \right.} \right\rangle$, with *θ*_*A*_ ∈ {0,2*π*} where |*e*〉 (|*l*〉) are early (late) pulses produced by the converter, can be transferred over the nLOS channel and measured at any arbitrary basis *θ*_*B*_ ∈ {0,2*π*} with high visibility, independently and simultaneously by the illuminated pixels of our SPDA analyzer.

For the demonstration, we use the experimental setup shown in Fig. 1 where the laser and Converter are assumed to be at sender’s (Alice’s) lab while the Analyzer and the camera at receiver’s (Bob’s) lab. The scattering surface is rotated at *φ* = 20° to ensure that photons detected by Bob are mostly due to scattering from the diffusive surface. By applying specific voltages at the converter (analyzer) Piezo actuator, specific phase values *θ*_*A*_ (*θ*_*B*_) are set. Figure 3c shows the variation of received photon counts at the illuminated pixels for Bob’s measurement basis *θ*_*B*_ = 0° when *θ*_*A*_ is varied from 0 to 2*π*. Similar variations are observed when Bob’s basis is changed to other arbitrary values, or when we scan the values of *θ*_*B*_ from 0 to 2*π* for a constant value of *θ*_*A*_ (not shown). In all cases, the average visibility was in the range of 0.9−0.95 for all the illuminated pixels which is comparable to the values reported in previous free-space quantum communication experiments involving time-bins^25,26,32,43,44^. The result verifies that our NLOS channel can be suitable for time-bin quantum communication experiments using scattered photons.

We emphasize that when used for quantum communication experiments, our SPDA sensor enhances the robustness of the QKD receiver, in addition to allowing nLOS operation. Firstly, the imaging information for each pixel can be used for tracking a transmitter beam that feeds into an active beam steering mechanism, or simply used to passively suppress background counts in processing, thus reducing complexity^26,32^. Secondly, the implementation of multiple photon detector pixels appears to be more robust against certain powerful quantum attacks. For example, the class of detector control attacks^45,46^ could be prevented by adopting the method presented in^47^. The efficiency mismatch type attacks^48^ – where an eavesdropper attacks by modifying the spatial modes of the incoming light – could be easily detected as changing the spatial mode will change the spatial distribution of detection events.

We note in passing that the sender-to-receiver distance (loss) in our experiment was ~2*m* (6 dB). However, for free-space line-of-sight quantum communication experiments, phase-coherence has been shown to be robust for much longer distances and higher losses (ex. 5000 km and 60 dB in ref. ^26^, 31 dB in ref. ^32^). The distance is ultimately limited by the dispersion properties of the medium and possibly the structure of the object, as it would cause the pulse width to widen and overlap with each other. Thus, the focus of this section was to investigate the robustness of coherence against scattering property of the channel.

### Low-light imaging in high photon background

**Fig. 4:**
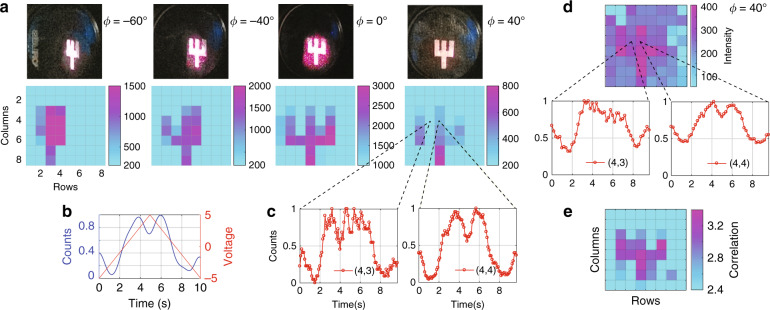
Enhancing the contrast of an image. **a** The top and bottom row show the illuminated object (size 4 mm x 3.6 mm) – with the scattering surface rotated at four different angles $\varphi \in \{ - 60^0, - 40^0,0^0, + 40^0\}$ – and the corresponding images observed in the camera. Only the events detected during the 0.6 ns window – centered around the middle-peak – were post-selected to form the image. **b** The variation of counts (normalized) as a function of the applied phase-signature. This is the expected count pattern – the reference pattern – for high signal-to-noise ratio scenario. **c** The observed pattern in response to the phase-signature for two neighboring pixels (4,3) and (4,4) for rotation *φ* *=* 40^0^. **d** Image captured by the SPDA imager in the low-light and noisy environment. The observed pattern at the same two neighboring pixels (4,3) and (4,4) are also shown. **e** The image reconstructed by cross-correlating the observed pattern with the reference pattern (see text and Methods section for more details)

It is important to note that the ability to reveal quantum coherence can be directly used to enhance the contrast of an image under a low-light and noisy environment. The main idea is as follows. An object is illuminated with photons prepared with a specific phase-signature that is maintained by the scattered photons. As the scattered photons are imaged by the SPDA analyzer, the detected count pattern should follow the applied phase-signature. Hence, correlating the phase-signature with the received count pattern, a scattered photon can be distinguished from a noise photon with enhanced signal-to-noise ratio. Here, we show a proof-of-principle demonstration of this concept. In order to bring coherence into the picture, a periodic ramp voltage (0.1 Hz, ±5 *V* peak-to-peak) was applied to the Converter piezoelectric actuator that encoded a periodic phase-signature pattern among the outgoing time-bin states. The count pattern at the SPDA pixels in response to this phase-signature was pre-characterized as shown in Fig. 4b. All the pixels followed roughly the same pattern and from now on, we shall refer to this as the reference pattern.

The experimental setup is similar to Fig. 1, but now an object is placed on the scattering surface and the laser focusing condition is adjusted such that a sharp image is observed at the SPDA camera. Figure 4a shows the conventional intensity-based images of the object for four different rotations of the scattering surface. From now on, we focus only on the case where the scattering surface was rotated to *φ* = 40^0^, ensuring that the specular reflection no longer reaches the camera, and the detected photons were only due to diffusive scattering. The coherent variation of intensity across the image in response to the applied phase is shown in Fig. 6 in Methods section.

First, we focus on two neighboring pixels (4,4) and (4,3). Figure 4c shows the variation of counts in these two pixels in response to applied phase-signature. These count patterns are hereafter referred to as observed pattern. The observed patterns appear distorted due to low SNR, however the correlation with the reference pattern is clearly visible. Since pixel (4,4) received more photons than (4,3) – due to the feature of the object – its pattern appears more correlated to the reference pattern. This particular feature has been exploited to enhance the contrast as described next.

In order to simulate a noisy low-light environment, the laser was attenuated to 0.8 photons per pulse and a lamp was placed in front of the camera to create a high level of background signals. The average number of noise photons was 50 per pixel per second while the average number of signal photons varied from 124 to 388 per second depending on the position of the pixels leading to a signal-to-noise ratio of 2.4−7.76. The scattering surface rotation was kept at *φ* = 40^0^. The image captured by the imaging array is shown in Fig. 4d. As expected, the presence of high background severely degraded the image contrast. The observed pattern at the same two pixels (4,3) and (4,4) are also included in Fig. 4d which shifted up due to the presence of high background photons, yet the correlation with the reference pattern is still visible. For each pixel, we calculated the correlation between the observed and reference pattern (see Methods section for more details). We have chosen a threshold of 2.4, and Fig. 4e shows only those pixels having a correlation higher than this threshold. The result is a reconstruction of the object image with much-enhanced contrast. We note that only photons from the SL and LS paths have been considered here. In other words, from the three peaks shown in Fig. 2, we only post-selected the detections from the middle-peak which required the timing data for each pixel. As a result, our approach is a combination of utilizing arrival times for coherence analysis, that can also be utilized for LIDAR applications.

We would like to elaborate on how our coherence-based imaging could complement the conventional intensity-based imaging. When intensity is used as a distinguishing parameter, the signal-to-noise ratio decreases linearly with an increase in noise or decrease in signal intensity. On the other hand, as shown in Fig. 3b, visibility – the measure of coherence – stays fairly constant with decreasing signal intensity. This means when the number of detected signal photon decreases, the relative amplitude of the count pattern decreases but the shape of the pattern stays fairly same. As a result, correlation with the reference pattern decreases very slowly. In fact, the scheme could be generalized by utilizing both the outputs of the Analyzer interferometer. In that case, the total intensity at the two output arms – as a function of applied-phase *θ* – *P*_±_(*θ*) can be written as

1 $$P_ \pm (\theta ) = S_ \pm (\theta ) + N/2$$

where *N* is the background noise, which is assumed equally distributed among the two outputs arms, while *S*_±_(*θ*) are the signal intensity at each output which are inverse (*π*-phase shifted) of each other. Consequently, the difference function $P_ + (\theta ) - P_ - (\theta )$, could theoretically cancel out any background noise and increase the signal-to-noise ratio manifold. In a preliminary study, we have already implemented this scheme and were able to distinguish a signal photon from the noise with noise intensity 10 times higher than signal intensity. However, finding a more sophisticated image-reconstruction method is out of this paper’s scope and will be presented elsewhere.

## Discussion

This work has demonstrated a novel and robust approach to transfer and recover quantum coherence via scattered photons by realizing a multimode, imaging time-bin interferometer equipped with a single-photon-detector-array sensor. Our quantum receiver achieves excellent temporal precision of ≈120 ps as well as the ability to spatially resolve the field-of-view with excellent time-bin visibility across the whole sensor area. Each pixel independently received the coherence from the spatial modes of the scattered photons. The maximum observed visibility was 95%, which remained within a <10% variation over a wide scattering angle range of –45° to +45°, recovered from photons through a scattering, non-line-of-sight channel. All these features have the potential to open up new avenues in many applications, including quantum communication around-the-corner, low-light and 3D imaging, background noise rejection, around-the-corner imaging^10^, velocity measurement^12^, LIDAR, object detection and identification, etc.

We demonstrated the application potential of our method by showing two potential applications, one is non-line-of-sight quantum communications, the other is enhancing the contrast of single-photon images. A more detailed and quantitative analysis of these applications will be given elsewhere. We believe our results will instigate further research on the application of coherence in quantum sensing, imaging, and communication and lead to novel areas of application.

## Materials and methods

### Detailed experimental setup

**Fig. 5:**
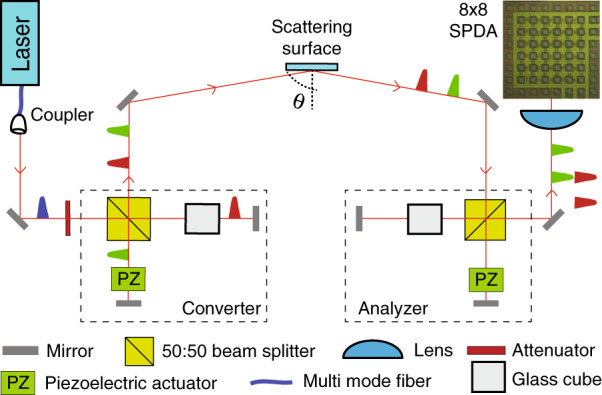
The experimental setup. The Converter creates two pulses separated by 0.6 ns. The path difference between the two short-long (SL) and long-short (LS) pulses can be adjusted using the piezoelectric actuator placed in the short arm. A glass cube is present in the long arm of both interferometers to make the interferometers balanced in the spatial domain. The Analyzer recombines the pulses coming via SL and LS paths and the output is studied by the 8 × 8 SPDA. During the initial alignment, the incident angle was *θ* = 25° (figure not to scale)

The experimental setup (Fig. 5) consists of two unbalanced Michelson interferometers (UMIs). The first UMI – Converter – creates the coherence while the second one – Analyzer – measures the coherence. The laser (Picoquant laser diode LDH 8-1596, PDL-800B driver) emits 697 nm, 300 ps multimode light pulses at a rate of 5 MHz. Lights coming out of the multimode fiber are collimated, attenuated to 0.3 photon per pulse and sent through the Converter. The path difference ∆_*C*_ between the long and short arms turns each incoming state into a coherent superposition of two time-bins separated by 0.57 ns. They are sent towards the scattering surface and the scattered photons are collected by the Analyzer. The path difference between the short and the long arm in the Analyzer is ∆_*A*_. A photon coming out of the Analyzer could have traversed one of the four possible paths: short-short (SS), short-long (SL), long-short (LS), and long-long (LL). If the path-differences in each UMI match, i.e., ∆_*C*_ ≈ ∆_*A*_, the probabilities of a photon coming via SL and LS paths become indistinguishable and result in interference. Piezoelectric crystals are placed in the short arm of each interferometer to adjust the path difference. The photons emerging from the Analyzer are focused into an SPDA containing 64 single-photon avalanche photodiodes. The pixels of the SPDA are arranged in a 8 × 8 row-column configuration, with a pitch of 75 µm. A focusing lens (Canon EF-S 18-200 mm f/3.5-5.6) is used to illuminate the desired range of pixels with its focal length set to about 60 mm, thus yielding an angular resolution of 0.07°, and a total angular field of 0.5°. The detection efficiency at each pixel was 12%.

### Compensation for spatial-mode distortion

A time-bin encoded photon entering an Analyzer with variable angle of incidence causes a lateral offset between the paths impinging at the exit beamsplitter^24,49^ causing degradation of interference visibility. Channel induced spatial-mode distortions further lower the interference quality. The root cause of this is the inherent asymmetry of the unbalanced Michelson interferometers. A compensation technique is to use a glass material with appropriate length and refractive index to create a virtual mirror closer to the beamsplitter. In this way, the interferometer, although asymmetric in time, becomes symmetric in the spatial-mode domain. We placed a 118-mm-long glass cube with refractive index 1.4825 at the long arm to match the distance between beamsplitter-to-virtual-mirror with the short arm (see^49^ for more details and the analytical formula). This compensation not only improves performance at higher AOI, but is also necessary to enable high interference visibility with a multimode beam.

### Imaging while observing photon interference

**Fig. 6:**
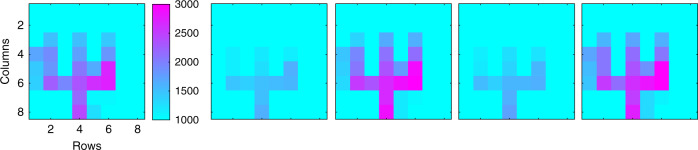
Image and interference observed simultaneously. Images for five different phase-instances are shown along with the coherent variation of intensity across the illuminated pixel. The visibilities varied in the range 0.85−0.95

The intensity of image at different instant of time is shown in Fig. 6. As the phase between time-bins is varied at the converter, the intensity varies across the whole image and the variation across all illuminated pixels is coherent, i.e., they vary in unison similar to Fig. 3c. The figure only shows events detected during the 0.6-ns window – centered around the middle-peak. The visibility was in the range 0.85−0.95.

### Correlation of patterns

**Fig. 7:**
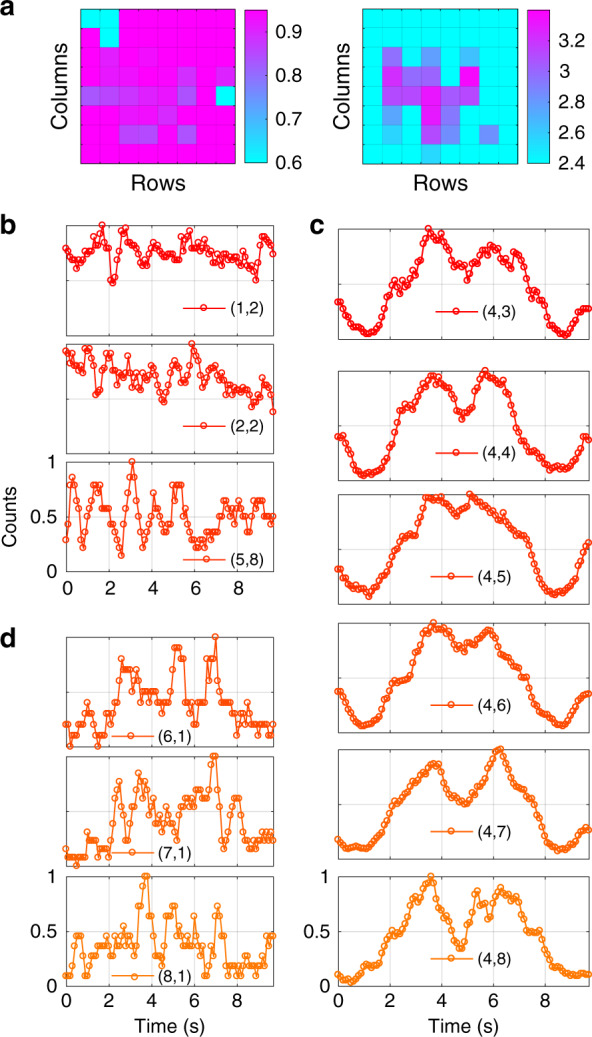
Visibility and patterns observed at different pixels of the SPDA imager. **a** Visibility across all the pixels (left) and the reconstructed image drawn from correlation (right). **b** Observed (normalized) pattern at the three defective pixels having high dark counts. The correlation with the reference pattern is lower than threshold. **c** Observed (normalized) pattern at the bottom six pixels of column four. The correlation with the reference pattern is higher than the threshold. **d** Observed pattern (normalized) at bottom three pixels of column one. The correlation with the reference pattern is between the two previous extremes but still lower than the chosen threshold. By plotting the pixels with correlation higher than the threshold, the image was reconstructed with enhanced contrast as shown in fig a (right)

Here we explain the method by which Fig. 4e was plotted. Figure 7a shows the same image (right) along with the observed visibility (left) across all the SPDA pixels. The visibility was calculated as *V* = (*I*_max_ − *I*_min_)*/*(*I*_max_ + *I*_min_) where *I*_max_ (*I*_min_) is the maximum (minimum) count in a phase-signature cycle. Pixel (1,1) was the trigger, and (1,2), (2,2), and (5,8) had very high dark counts. The normalized observed pattern at these three defected pixels are shown in Fig. 7b where no correlation with the reference pattern was visible. Figure 7c shows the observed (normalized) pattern at the bottom six pixels of column 4. The resemblance with the reference pattern was quite pronounced. On the other hand, Fig. 7d shows the observed (normalized) patterns for the bottom three pixels of column 1 that detected relatively fewer number of photons and are much more distorted. They are in the middle of the two previous extremes and the correlation value with the reference pattern for them lies in the middle. Before calculating the cross-correlation, both the reference and observed patterns were shifted downward by 0.5 for the ease of analysis.

### The single-photon-detector-array sensor

**Fig. 8:**
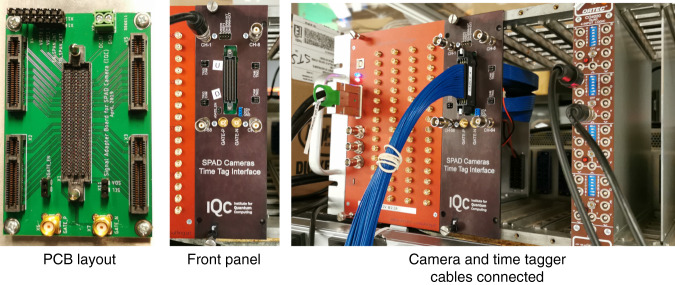
Interface board. The camera output (64 LVDS pairs) are linked to an interface board, and divides the signals to 4 blocks of 16 LVDS pairs, as well as provides control signal inputs. The 4 signal blocks are directly interfaced with a high-speed time-tagger that can handle 64 input channels, up to 1 GTag/s

The SPDA camera is composed of 64 single-photon avalanche diodes arranged in a square pattern with 8 rows and 8 columns. Each row and column has eight 30 µm diameter detectors with X and Y pitch equal to 75 µm. Each photon arriving on each pixel can be individually detected with a precision of 100 ps, and over 2 billion photons per second can be detected across the array. This places stringent requirements on the time-tagging electronics which cannot be met by existing products. As a result, we have developed a new adapter board to interface the SPDA camera with our time-tagging device. The combination of the SPAD camera, time-tagging electronics and the developed adapter board with the corresponding software provided a unique quantum sensor with exquisite temporal and spatial resolution. The electrical output from the camera (Samtec Cable, SEAC-30-08-XX.X-TU-TU-2) contains 64 LVDS detection signal from the pixels along with other control signals. The developed adapter board translates the 64 LVDS camera outputs into four groups of 16 LVDS channels going into the time-tagger (Universal quantum devices) as shown in Fig. 8. As a result, time stamps from the camera can be read by the tagger at a rate of approximately 1 GTag/s through an external-PCI-express interface. The adapter board provides the option to sacrifice upto four pixels (1, 8, 58, and 64) to be used as external trigger. In this work only one trigger was used. When triggering is not required, the pixels can function as normal detectors. The average dark count rate was 35 cps and the deadtime was 150 ns.
